# Clinical Efficacy of Extracellular Vesicle Therapy in Periodontitis: Reduced Inflammation and Enhanced Regeneration

**DOI:** 10.3390/ijms25115753

**Published:** 2024-05-25

**Authors:** Miljan Puletic, Gordana Velikic, Dusan M. Maric, Gordana Supic, Dusica L. Maric, Nikola Radovic, Stevan Avramov, Danilo Vojvodic

**Affiliations:** 1 Faculty of Stomatology Pancevo, University Business Academy, 26101 Pancevo, Serbia; miljenko.puletic@gmail.com (M.P.); ducamaric@gmail.com (D.M.M.); nikolaradovic.dr@gmail.com (N.R.); stevan@ibiss.bg.ac.rs (S.A.); 2 Department for Research and Development, Clinic Orto MD-Parks Hospital, 21000 Novi Sad, Serbia; 3 Hajim School of Engineering, University of Rochester, Rochester, NY 14627, USA; 4 Institute for Medical Research, Military Medical Academy, 11000 Belgrade, Serbia; gogasupic@gmail.com (G.S.); vojvodic.danilo@gmail.com (D.V.); 5 Medical Faculty of Military Medical Academy, University of Defense, 11000 Belgrade, Serbia; 6 Department of Anatomy, Faculty of Medicine, University of Novi Sad, 21000 Novi Sad, Serbia; 7 Institute for Biological Research “Sinisa Stankovic”, National Institute of the Republic of Serbia, University of Belgrade, 11060 Belgrade, Serbia

**Keywords:** exosomes, periodontitis, cytokines, regenerative medicine, extracellular vesicles

## Abstract

Periodontitis, a prevalent inflammatory condition, affects the supporting structures of teeth, leading to significant oral health challenges. Traditional treatments have primarily focused on mechanical debridement, antimicrobial therapy, and surgery, which often fail to restore lost periodontal structures. Emerging as a novel approach in regenerative medicine, extracellular vesicle (EV) therapy, including exosomes, leverages nano-sized vesicles known for facilitating intercellular communication and modulating physiological and pathological processes. This study is a proof-of-concept type that evaluates the clinical efficacy of EV therapy as a non-surgical treatment for stage I–III periodontitis, focusing on its anti-inflammatory and regenerative potential. The research involved seven patients undergoing the therapy, and seven healthy individuals. Clinical parameters, including the plaque index, bleeding on probing, probing depth, and attachment level, were assessed alongside cytokine levels in the gingival crevicular fluid. The study found significant improvements in clinical parameters, and a marked reduction in pro-inflammatory cytokines post-treatment, matching the levels of healthy subjects, underscoring the therapy’s ability to not only attenuate inflammation and enhance tissue regeneration, but also highlighting its potential in restoring periodontal health. This investigation illuminates the promising role of EV therapy in periodontal treatment, advocating for a shift towards therapies that halt disease progression and promote structural and functional restoration of periodontal tissues.

## 1. Introduction

Periodontitis, a multidimensional inflammatory condition, primarily targets the supporting structures of the teeth, leading to formidable oral health challenges, including tooth mobility and, ultimately, tooth loss (Table 1). Characterized by the persistent inflammation and degradation of the periodontal ligament and alveolar bone, periodontitis establishes a nexus with systemic health issues and autoimmune diseases like cardiovascular diseases, rheumatoid arthritis, and diabetes [1,2,3,4]. Traditionally, the management of periodontitis has pivoted around mechanical debridement, antimicrobial therapy, and surgical interventions aimed at mitigating the inflammatory response and inhibiting the progression of the disease [5]. Nevertheless, these conventional approaches seldom restore the lost periodontal structures, an aspect that necessitates the exploration of innovative therapeutic modalities.

In the booming field of regenerative medicine, extracellular vesicle (EV) therapy has emerged as a promising candidate for treating periodontitis, traversing beyond the mere mitigation of symptoms to potentially facilitating the regeneration of damaged periodontal tissues [7,8]. Exosomes, nano-sized EVs, are renowned for their essential role in intercellular communication [9]. They transport many biomolecules, including proteins, lipids, and nucleic acids, between cells, modulating various physiological and pathological processes [10]. Exosomes can carry and deliver interleukins [ILs] to target cells, thus modulating immune responses and facilitating tissue repair and regeneration [11]. Although periodontitis is not typically categorized as an “immunomodulatory disease” per se, its progression and pathology are deeply intertwined with immunomodulatory processes [12]. In periodontitis, ILs play a dual role: they contribute to inflammation and tissue destruction by mediating immune responses and bone resorption. Elevated levels of specific ILs in response to periodontal pathogens exacerbate this condition’s chronic inflammation and tissue damage. However, they can also serve as potential therapeutic targets for controlling the disease’s progression [13,14]. Additionally, the response of cells to ILs can lead to the release of exosomes, which further influences the regenerative process [11]. This synergistic relationship is a key area of research in regenerative medicine, as understanding and harnessing these interactions could lead to novel therapeutic strategies that circumvent the limitations of conventional treatment modalities for a wide range of diseases and injuries, including periodontitis [15].

The deployment of EV therapy in periodontitis treatment transcends the conventional therapeutic boundaries, offering a multivariant approach that addresses not only the symptomatic manifestations of the disease, but also targets the underlying pathophysiological mechanisms [16]. Our research has confirmed the potent capability of EVs, including exosomes and their cargo, to attenuate inflammation within the periodontal milieu, disrupting the inflammatory cascade that propels periodontal degradation. Furthermore, the EVs demonstrated a remarkable propensity to promote tissue regeneration, reducing the periodontal pocket depth and restoring the disrupted periodontal architecture [17].

This innovative approach is well-suited to developing advanced therapeutic strategies that connect EVs’ anti-inflammatory and regenerative capabilities, providing a comprehensive treatment modality for periodontitis. As we delve deeper into the mechanisms underpinning the therapeutic effects of EV treatment, we navigate a paradigm shift in periodontal therapy, where the focus is on arresting the disease progression and reinstating the structural and functional integrity of the periodontal apparatus.

The following sections of this paper outline the methodologies employed in our research, and explore the potential implications of these findings in the broader context of periodontal therapy and regenerative medicine as clinical evidence of the mechanistic pathways through which EVs exert their anti-inflammatory and regenerative effects. The synthesis of scientific inquiry and innovative treatment modalities, as exemplified by the deployment of this therapy, signifies a monumental stride towards redefining the therapeutic approach to periodontitis and enhancing the quality of life for the affected individuals.

In light of the latest recommendations from the Minimal Information for Studies of Extracellular Vesicles 2023 (MISEV2023) [18], we have updated the terminology in our manuscript to align with the current scientific standards. Instead of the term “exosomes”, we will primarily use “extracellular vesicles” or “EVs”, which encompasses a broader range of vesicular entities as defined by their biogenesis, size, and content. This change ensures accuracy and clarity in describing our research findings, as the term “EVs” includes all particles released from cells, irrespective of their specific routes of biogenesis or size. However, we will specifically refer to “exosome cargo” when discussing materials encapsulated within EVs that exhibit characteristics typical of exosomes, such as specific biomarker profiles, according to MISEV guidelines. This terminology refinement enhances the precision of our discourse and aligns our study with contemporary vesicle nomenclature and classification practices.

## 2. Results

### 2.1. Demographic and Clinical Characteristics

The study included 14 participants, seven subjects—four males and three females with generalized periodontitis stages I–III, and seven healthy subjects, also four males and three females. The optimal number of subjects for this pilot study was determined through statistical analyses utilizing the G Power 3.0.10 program [19], ensuring a study power greater than 98% at a significance level of 0.05.

The clinical characteristics are given in Table 2. The post-treatment values indicate statistically significant reductions in periodontal disease indicators (*p* < 0.05), underscoring the efficacy of the treatment approach.

### 2.2. The Cytokines Levels—Basic Analyses

One of the primary challenges in managing active periodontitis involves identifying active sites characterized by ongoing tissue destruction and likely increased cytokine secretion in gingival crevicular fluid (GCF) [20]. Before treatment, these sites exhibited elevated levels of various cytokines, indicative of heightened inflammatory activity. Our data reveal that active sites before treatment have significantly higher cytokine concentrations than the post-treatment inactive sites, as detailed in Table 3 and Table 4. This distinction underscores the critical role of cytokines in driving the pathogenic processes at these sites and highlights the effectiveness of EV-based periodontal treatment in reducing inflammation and cytokine levels.

Skewness quantifies the asymmetry of a distribution, with positive and negative values indicating a tail extending to the right or left, respectively. Kurtosis measures the tail heaviness of a distribution, where higher values indicate a greater presence of outliers. We employed the Shapiro–Wilk test to assess the normality of our data. Post-treatment changes in skewness and kurtosis imply that the treatment had differential impacts on the distribution of cytokine levels, with the noteworthy observation that many cytokines exhibited non-normal distributions after EV treatment.

### 2.3. The Cytokines Levels Advanced Analyses

**Figure 1:**
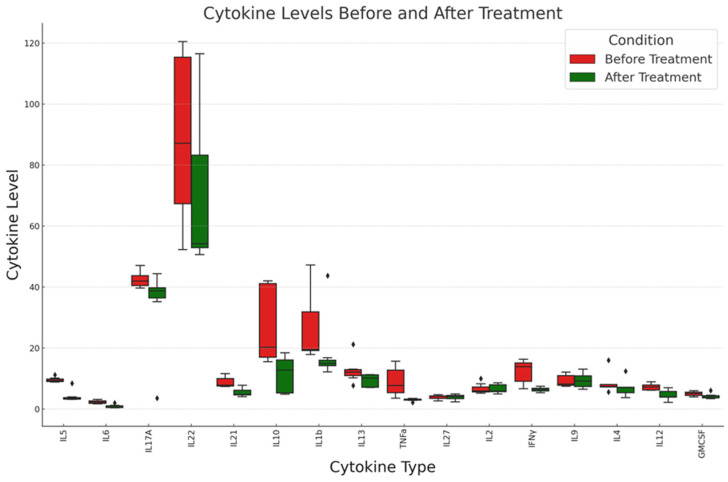
Comparative analysis of cytokine levels before and after treatment: This boxplot illustrates the distribution of various cytokine levels in patients before (red) and after (green) EV treatment regimen. The vertical axis represents the cytokine levels, while the horizontal axis lists the different cytokines measured, and diamonds the outliers. See text for details.

Guided by descriptive analyses and the confirmation of normal distribution, as detailed in Table 3 and Table 4, we chose sophisticated statistical techniques to analyze cytokine dynamics following treatment. Figure 1 provides a graphical comparison of cytokine levels before and after treatment. Table 5 presents the outcomes of paired statistical tests comparing cytokine levels pre- and post-treatment. Additionally, Table 6 presents the significant cytokine levels before and after the treatment per each patient.

Total amounts of IL22, IL27, IL2, IL9, IL4, IL1β, IL13, and GMCSF in active sites (before treatment) were lower than in their inactive sites (6 weeks after the treatment), but the differences did not reach the level of significance (*p* < 0.05). It is important to consider the clinical significance of these findings, in addition to their statistical significance. Even if statistical significance is not achieved, the direction and magnitude of change in cytokine levels can offer insights into the biological impact of EV therapy, which is detailed more in the following sections.

Cytokines IL5, IL6, IL17A, IL10, and IL21 showed significant changes in levels after treatment, as indicated by the *p*-values being below 0.05. Due to their non-normal distribution, these cytokines were analyzed using the Mann–Whitney U test.

Cytokines TNFα, IL27, IL2, IFNγ, IL9, IL12, and GMCSF were analyzed using the paired *t*-test. Among them, TNFα, IFNγ, and IL12 showed significant changes after the treatment.

### 2.4. Analysis of Treatment Efficacy

The applications of the Mann–Whitney U tests and paired *t*-tests in our analyses played a critical role in substantiating the efficacy of EV therapy. Specifically, the Mann–Whitney U tests revealed statistically significant reductions in levels of key pro-inflammatory cytokines, such as IL-5, IL-6, and IL-17A, post-treatment in the EV therapy group [7,21,22]. These results indicate a profound anti-inflammatory effect attributed to EV therapy, highlighting its potential to disrupt the pathological inflammatory processes characteristic of periodontitis. Similarly, the paired *t*-tests demonstrated significant changes in cytokine dynamics, further validating the regenerative capacity of the therapy. For instance, the significant reduction in TNF-α levels post-treatment underscores EV therapy’s role in mitigating tissue destruction and promoting periodontal health. Collectively, these statistical analyses underscore the dual anti-inflammatory and regenerative potential of the therapy, offering a promising therapeutic pathway for the management of periodontitis [23,24].

#### Healthy Subjects as a Baseline for Normal Cytokine Levels

We present the calculated descriptive statistical values for healthy subjects’ cytokine levels in Table 7. 

The statistical tests comparing cytokine levels between healthy subjects and subjects with periodontitis before treatment yielded the *p*-values in Table 8. According to Table 8, cytokines IL5, IL6, IL17A, IL21, IL10, IL1β, IL13, TNFα, IFNγ, IL12, and GMCSF show statistically significant differences between healthy subjects and subjects with periodontitis before treatment. This indicates elevated or altered cytokine levels in periodontitis patients compared to healthy individuals, reflecting the inflammatory nature of periodontitis. Cytokines IL22, IL27, IL2, and IL9 did not show statistically significant differences in this comparison. For IL22 and IL4, the *p*-value is close to the threshold for significance, suggesting a possible difference that might become clearer with a larger sample size or further study.

Table 9 provides the statistical tests comparing cytokine levels between healthy subjects and subjects with periodontitis six weeks post-treatment.

None of the cytokines show a statistically significant difference between healthy subjects and subjects with periodontitis at the six-week follow-up, given the conventional alpha level of 0.05 for statistical significance. This suggests that, based on the cytokine levels analyzed, there is no statistically significant difference between the post-treatment levels in subjects with periodontitis after the EV therapy and the levels in healthy subjects for the cytokines tested.

## 3. Discussion

### 3.1. The Retrospective Analysis of Results

The study investigates using EV therapy as a potential therapeutic approach for periodontitis. It is a proof-of-concept study, meaning that focus is on providing an evidence that EV therapy produces the expected biological and immunological response in periodontitis patients. Periodontitis is a complex inflammatory condition characterized by the progressive destruction of the periodontal ligament and alveolar bone, leading to tooth mobility and eventual loss. The pathogenesis of periodontitis is closely linked to the dysregulation of the host immune response, which involves the production of various inflammatory cytokines. These cytokines are crucial in orchestrating the inflammatory cascade, ultimately contributing to tissue damage and impaired regeneration. EVs facilitate intercellular communication by transporting various biomolecules, including proteins, lipids, and nucleic acids. In the context of periodontitis, EVs have been proposed as a promising therapeutic modality due to their ability to modulate immune responses, attenuate inflammation, and promote tissue regeneration. See Table 10 for summary of the key findings.

The results of this study demonstrate a significant reduction in the levels of pro-inflammatory cytokines IL-5, IL-6, IL-17A, IL-10, and TNF-α, after the therapy. These cytokines play crucial roles in the pathogenesis of periodontitis, and are implicated in various processes, such as inflammation, bone resorption, and tissue destruction (see Table 9).

IL-5 is a cytokine involved in the activation and recruitment of eosinophils, which contribute to inflammation and tissue damage in periodontitis [25]. IL-6 is a pleiotropic cytokine that plays a role in the acute phase response and can stimulate osteoclast differentiation, leading to bone resorption. IL-6 has important effects in response to microbial insults, acting not only as an anti-inflammatory agent, but also as a proinflammatory agent when the inflammatory process becomes chronic [26,27,28]. IL-17A is a pro-inflammatory cytokine produced by Th17 cells and is associated with the recruitment of neutrophils and the promotion of osteoclastogenesis [29,30]. IL-10, while traditionally considered an anti-inflammatory cytokine, has been found to have complex roles in periodontitis [31,32]. TNF-α is a potent pro-inflammatory cytokine that can induce the production of other inflammatory mediators, promote osteoclastogenesis, and contribute to tissue destruction [33,34].

The observed reductions in these cytokine levels after the treatment suggest a potential anti-inflammatory effect, which may contribute to the attenuation of the inflammatory cascade and the subsequent mitigation of tissue damage in periodontitis. The modulation of these cytokines may create a more favorable environment for tissue regeneration by reducing the inhibitory effects of excessive inflammation on the regenerative processes.

It is important to note that the study also analyzed other cytokines, such as IL-22, IL-27, IL-2, IL-9, IL-4, IL-12, and GM-CSF, which did not show significant changes after treatment. These cytokines have diverse functions in the immune response, ranging from promoting inflammation to regulating tissue repair and regeneration. The lack of significant changes in these cytokines may indicate the complex interplay between various cytokine pathways and the specific mechanisms targeted by EV therapy.

The distribution patterns of cytokine levels before and after the treatment for periodontitis revealed intriguing differences. While some cytokines maintained their original distribution, many exhibited non-normal distributions following the therapy. This phenomenon can be attributed to a multitude of factors intricately woven into the nature of the research, the disease, the treatment modality, and the characteristics of the cytokines themselves [35,36,37,38]:

1. Individuals may respond diversely to EV therapy due to genetic and epigenetic variations, environmental influences, degree of disease, and lifestyle factors. These differences can lead to varying extents of cytokine modulation post-treatment, contributing to the non-normal distribution observed in specific cytokines after therapy. The individual variability in the biological response to the treatment can result in a wide range of cytokine levels, deviating from a normal distribution;
2. Periodontitis involves complex inflammatory processes with multiple pathways and feedback mechanisms. EV therapy may selectively target specific pathways, leading to significant changes in certain cytokines but not others. This targeted effect can cause alterations in the distribution of cytokine levels, as some may be directly affected by the therapy, while others are indirectly influenced or remain unchanged;
3. Cytokines regulate each other through intricate networks of positive and negative feedback loops. The modulation of one cytokine can initiate a cascade of effects on others, potentially resulting in a shift in their distribution patterns post-treatment. For instance, reducing pro-inflammatory cytokines might lead to an increase in anti-inflammatory cytokines, altering the overall cytokine profile and its distribution;
4. The stage of periodontitis and the degree of inflammation can influence the initial distribution of cytokines. EV therapy’s effectiveness in reducing inflammation and promoting tissue regeneration may not be uniform across all cytokines or disease stages, contributing to differences in their distributions before and after treatment;
5. The methods employed to measure cytokine levels and the analytical techniques used to assess their distribution can contribute to the observed differences. Variability in sample collection, handling, size, and analysis can impact the measured cytokine levels and their statistical distribution;
6. Not all biological data follow a normal distribution. Many physiological and biochemical parameters, including cytokine levels, may naturally follow skewed distributions due to the biological mechanisms governing their production and clearance. The treatment may shift cytokine levels closer to or further from a normal distribution, depending on how it affects these underlying biological mechanisms. Intriguingly, the normal distribution of cytokine levels for most cytokines under investigation after treatment closely resembles that observed in healthy subjects.

#### Unveiling the Complex Nature of Periodontitis: A Comparison with Baseline Inflammatory Marker Levels in Healthy Subjects

The comparison with healthy subjects’ results contributes to the evaluation of EV therapy’s efficacy in modulating the immune response towards a profile more closely resembling that of healthy individuals. Note that we are investigating the local immune response, and that normalization refers to local immune homeostasis. The normalization of some cytokine levels in the six-week follow-up post-treatment compared to the significant differences observed before treatment suggests that the therapy may have a beneficial effect in modulating the immune response towards a healthier profile. However, for a complete treatment assessment, comparing the post-treatment levels with both the healthy and pre-treatment levels is crucial to understanding the full impact of the therapy. Note that cytokines with statistically significant differences pre- and post-treatment are not entirely the same when comparing pre-treatment vs. healthy and post-treatment vs. healthy, yet the post-treatment cytokine levels are closer to normal (healthy) levels. Suppose we disregard the potentially small sample size influence, considering that this is a proof-of-concept study for evidence that EV therapy produces the expected biological and immunological response in periodontitis patients. In that case, this suggests several important points regarding the complex nature of periodontitis and the efficacy of the therapy.

Variability in inflammatory response: Periodontitis triggers an inflammatory response that varies among individuals. The cytokines with significant differences in the pre-treatment phase reflect the active inflammation and immune response to the periodontal disease. Not all cytokines may be elevated in all patients, indicating the heterogeneity of the inflammatory response in periodontitis.

Targeted effect of EV therapy: EV therapy appears to modulate specific cytokines, bringing their levels closer to those observed in healthy individuals. This suggests that the therapy may specifically target certain pathways of inflammation and immune response. The differences in which cytokines show significant changes pre- and post-treatment might reflect the therapy’s specific biological targets.

Resolution of inflammation: The reduction in levels of specific pro-inflammatory cytokines post-treatment, even if they are not the same ones identified in the pre-treatment comparison, suggests a general trend toward the resolution of inflammation. This could indicate that while the therapy directly influences specific cytokines, others may decrease as a secondary effect due to the overall reduction in periodontal inflammation.

Complex interactions in immune modulation: The immune system is highly complex and involves numerous interactions between different cells and cytokines. The therapy might directly affect specific cytokines which, in turn, influence the levels of others through the intricate network of immune regulation. This could explain why some cytokines which were not significantly different pre-treatment compared to healthy subjects became normalized post-treatment.

### 3.2. EV Therapy Benefits

The rationale for employing EVs in periodontitis treatment is founded on their distinct properties and therapeutic advantages. Being nano-sized, many EVs, including exosomes, facilitate intercellular communication by transporting proteins, lipids, and nucleic acids between cells, a process pivotal in numerous biological functions.

In tackling periodontitis, EVs bring forth several benefits:

1. Immunomodulatory effects: EVs can carry immunomodulatory molecules, including cytokines and growth factors, crucial in restoring immune balance. This is particularly beneficial in periodontitis, where an imbalance in immune response leads to chronic inflammation and tissue destruction. By modulating the production and activity of both pro-inflammatory and anti-inflammatory cytokines, EVs can help re-establish local immune homeostasis;
2. Tissue regeneration: They are integral to tissue repair, and can convey biomolecules that stimulate cell proliferation, differentiation, and regeneration. This capability is invaluable in periodontitis, as it helps heal damaged periodontal ligaments and alveolar bone;
3. Targeted delivery and safety: EVs offer a platform for targeted therapy by being engineered to carry specific therapeutic molecules directly to affected tissues, enhancing treatment efficacy and minimizing side effects. Their natural origin contributes to low immunogenicity and biocompatibility, reducing the likelihood of adverse immune reactions.

These attributes underscore EVs’ therapeutic potential in periodontitis, supported by their ability to transport biomolecules pertinent to disease pathogenesis and regeneration [39,40,41]. Additionally, EVs derived from mesenchymal stem cells (MSCs), known for their regenerative and immunomodulatory capabilities, could amplify the therapeutic benefits in treating periodontitis.

### 3.3. EV Therapy Potential Mechanisms

EVs exert their immunomodulatory and regenerative effects through various mechanisms, many of which are still being elucidated. One key mechanism involves the transfer of bioactive molecules, such as proteins, lipids, and nucleic acids, from the exosomes to target cells. These biomolecules can modulate intracellular signaling pathways, gene expression, and cellular functions within the recipient cells.

In the context of immune modulation, exosomes can carry immunoregulatory molecules, including lipids, proteins, and nucleic acid cargo, such as microRNAs (miRNAs), mRNAs, long non-coding RNAs (lncRNAs), and circular RNA. Among non-coding RNAs involved in post-transcriptional regulation of gene expression, exosomal miRNAs are the most abundant exosomal cargo molecules. These regulatory molecules can influence the activity and differentiation of various immune cells, such as T, B, and antigen-presenting cells. For example, exosomes derived from MSCs have been shown to carry anti-inflammatory molecules that can suppress the production of pro-inflammatory cytokines and induce the generation of regulatory T cells, thereby dampening excessive inflammatory responses [42,43].

Regarding tissue regeneration, EVs can facilitate the repair and regeneration of periodontal tissues by delivering growth factors, signaling molecules, and genetic materials to target cells. Exosomes derived from stem cells or other regenerative cell types have been found to promote cell proliferation, migration, and differentiation, and stimulate angiogenesis and extracellular matrix remodeling, all of which are crucial processes in tissue regeneration. Additionally, exosomes may modulate the activity of resident stem/progenitor cells within the periodontal tissues, enhancing their regenerative potential.

Moreover, EVs can exert antimicrobial effects by carrying antimicrobial peptides or modulating the immune response against periodontal pathogens. This aspect could contribute to disrupting the inflammatory cascade these pathogens initiate, creating a more favorable environment for tissue regeneration.

It is important to note that the specific mechanisms by which EVs modulate the immune response and promote tissue regeneration may vary depending on the EVs’ source and their cargo and the target cells and tissues involved. Further research is needed to fully elucidate these mechanisms and to optimize EV-based therapies for periodontitis and other regenerative conditions.

### 3.4. Clinical Implications

The application of EV therapy in periodontitis presents significant clinical opportunities by demonstrating how EVs can influence key cytokines like IL-5, IL-6, IL-17A, IL-10, and TNF-α. This insight into their mechanism suggests the potential for EV therapy to attenuate inflammation and enhance tissue regeneration by:

1. Reducing inflammatory responses: lowering levels of pro-inflammatory cytokines through EV therapy indicates a potential for dampening the inflammatory cascade, contributing to tissue damage in periodontitis;
2. Immune system modulation: EVs’ ability to adjust the function of immune cells such as T, B, and antigen-presenting cells can help restore immune homeostasis and foster an environment conducive to tissue repair;
3. Enhancing tissue regeneration: by delivering regenerative signals and biomolecules to target cells, EVs can play a critical role in healing damaged periodontal tissues.

A possible bias resulting from the small sample size and the lack of a placebo group or a negative group of untreated periodontitis patients in our study should be acknowledged (detailed in Section 3.5). However, our proof-of-concept study shows clinical evidence that EV therapy produces the expected biological and immunological responses in periodontitis patients. Proof-of-concept studies are typically not conducted in large study groups, and are not intended to demonstrate the effectiveness within a population; rather, they are intended to show a significant positive effect and interpret the results, which is more possible with a smaller sample size. Another reason for a smaller sample size is that an investigational treatment could potentially elicit pathophysiologic effects. As this preliminary study found significant differences before and after treatment, and between treated patients and untreated healthy controls, future studies on a larger number of patients will provide even more direct answers to the potential applications of EV treatment in periodontitis patients. Nevertheless, proof-of-concept studies generally have more latitude in statistical requirements, but provide evidence that a new treatment is likely to be successful in later stages with larger studies. The changes in the normality of cytokine distributions pre- and post-treatment potentially highlights the importance of personalized medicine approaches in managing periodontitis, and should encourage further research into identifying predictors of treatment response.

EV therapy could serve as an adjunct to traditional treatments, offering a personalized, less invasive option that mitigates inflammation, promotes tissue regeneration, and potentially reduces the need for surgical interventions [44,45]. This approach not only aims to improve patient outcomes, but also addresses the intricate relationship between the host immune response, nitric oxide (NO) production, and periodontal pathogens [46]. Inflammation and NO production are closely linked to the progression of periodontitis, with elevated NO levels associated with tissue damage. EV therapy’s capacity to modulate cytokine levels could lead to reduced NO production, mitigating its detrimental effects and fostering a conducive environment for periodontal repair. Additionally, by potentially disrupting the inflammatory response initiated by periodontal pathogens and exhibiting antimicrobial properties, the therapy offers a comprehensive strategy against periodontitis [8].

Recent studies have highlighted the significant role of oxidative stress markers such as Malondialdehyde (MDA) and Superoxide Dismutase (SOD) in peri-implant crevicular fluid (PICF), noting their correlation with clinical parameters like probing pocket depth (PPD) and bleeding on probing (BOP) [47]. This aligns with the known pathophysiological processes in periodontitis, where increased oxidative stress contributes to tissue degradation, although [47] does not report significant differences in oxidative stress markers between healthy and diseased peri-implant sites, suggesting that these markers alone may not suffice to differentiate health from disease states. However, the established presence of oxidative stress in periodontal diseases suggests a potential target for therapeutic intervention. In this context, EV therapy for periodontitis could be pivotal. EVs are known for their ability to modulate inflammation and tissue regeneration, properties that could be harnessed to influence oxidative stress pathways negatively impacted in periodontal disease. EV therapy could potentially deliver antioxidant enzymes like SOD directly to periodontal sites, or carry other antioxidative agents to reduce oxidative stress and improve tissue recovery. Moreover, EVs could be engineered to enhance their antioxidative properties, thereby offering a dual approach to reduce inflammation and manage oxidative stress in periodontal tissues. Including oxidative stress markers in evaluating EV therapy effectiveness could also provide a more comprehensive analysis of its benefits beyond traditional clinical measures. By assessing changes in markers like MDA and SOD pre- and post-EV therapy, researchers could gain insights into the biochemical impacts of the treatment, potentially leading to optimized therapeutic strategies.

Healthy subjects can offer a baseline for normal cytokine levels, which helps us understand how close the treated patients’ levels come to what might be considered “normal” or “healthy” post-treatment. Understanding which cytokines are most significantly affected by therapy can guide the development of targeted treatment strategies. It also underscores the importance of personalized medicine approaches in managing periodontitis. The cytokines with significant differences before treatment highlight key areas of immune response and inflammation that are affected by periodontitis. Comparison of those levels is crucial for evaluating the therapy’s potential in not just mitigating disease symptoms, but also in restoring periodontal health to a state resembling that of individuals without periodontitis. This comparison might help identify which cytokines are most effectively normalized by the therapy and which remain elevated, suggesting areas for potential improvement in treatment protocols or highlighting specific cytokines as targets for adjunct therapies.

#### Raising the Bar in Periodontal Regenerative Medicine

In the evolving field of regenerative medicine for periodontitis, a range of innovative therapies have demonstrated the potential to promote tissue regeneration and enhance the outcomes of conventional treatments [48]. Among these, Platelet-rich plasma (PRP) and Platelet-rich fibrin (PRF), both derived from the patient’s blood and rich in growth factors, have been shown to stimulate tissue healing and bone regeneration effectively [49,50]. Similarly, stem cell therapy, particularly the use of mesenchymal stem cells (MSCs) harvested from bone marrow or adipose tissue, and tissue engineering techniques involving bioactive scaffolds that deliver cells, drugs, or proteins directly to the sites of damage, have garnered attention [51,52]. These scaffolds support cellular adhesion and proliferation, facilitating the integration of new tissue into existing structures. The application of growth factors such as bone morphogenetic proteins (BMPs) further represents an advanced therapeutic option by enhancing bone regeneration and periodontal tissue repair [53,54]. In addition, recent advancements in light-based therapies, such as photobiomodulation, have shown promise in regenerative medicine by enhancing cellular activity and tissue repair processes through non-invasive light application [55]. This technique complements the regenerative strategies by potentially reducing inflammation and accelerating healing processes, broadening the scope of treatment options available for periodontitis. EV therapy, however, stands out due to its unique advantages, offering a potentially superior choice for conditions like periodontitis. EVs naturally occur in the body, and are integral to cellular communication and tissue homeostasis, thus offering inherent biocompatibility and reducing the risk of adverse immune reactions—a common concern with therapies involving foreign materials or genetically modified cells. EVs’ capability to deliver a diverse array of bioactive molecules directly to targeted cells allows for precise modulation of the local environment, promoting healing and regeneration more effectively than other therapies. Additionally, the administration of EVs is less invasive, can be customized to enhance regenerative properties or carry specific therapeutic agents, and supports synergistic effects by delivering multiple therapeutic molecules simultaneously.

### 3.5. Discussion of Limitations

While this study’s results are promising, it is important to remember that it is a proof-of-concept study, and acknowledge certain design and methodology limitations that could influence the interpretation of the results.

The relatively small sample size may limit the generalizability of the findings. A larger, multicentric study would enhance the results’ statistical power and external validity, enabling a more comprehensive understanding of EV therapy’s impact across diverse populations. Additionally, the study did not investigate the long-term effects of the therapy or the potential mechanisms underlying the observed changes in cytokine levels. The study’s cross-sectional design limits our ability to draw definitive causal relationships between the therapy and observed clinical improvements. A longitudinal study design would provide stronger evidence by tracking changes over time and establishing a clearer cause–effect relationship. The absence of blinding and randomization in our study setup may introduce biases. Note that the healthy subjects serve as a reference point rather than a control group in the traditional sense. The lack of a control group receiving a placebo or standard care in the original study design is a limitation that should be acknowledged. Future studies could include both a healthy control group and a placebo-treated group with periodontitis to comprehensively evaluate the therapy’s efficacy. Without these controls, the placebo effect cannot be ruled out, potentially skewing the perceived efficacy of the treatment. Future studies should incorporate double-blind, randomized controls to minimize bias and provide a more robust evaluation of the therapy’s effectiveness. The study was conducted with a relatively homogenous population in terms of demographic and genetic backgrounds, which may limit the generalizability of the findings across diverse populations. Future studies should include a broader demographic, encompassing varied ethnicities, ages, and genetic backgrounds, to ensure that the results apply to a broader patient base. This diversity is crucial for understanding the differential impacts of EV therapy on periodontitis, which may vary significantly across different groups due to genetic, environmental, and lifestyle factors. The study did not account for the variability in EVs preparations, which might affect the consistency and reproducibility of the therapeutic outcomes. Standardizing the characterization of EV extraction and preparation methods is crucial for ensuring that the results are attributable to the therapy itself, rather than variations in the biological product.

Measuring clinical parameters and cytokine levels alone may not capture the full spectrum of periodontal health improvements or underlying biological changes. Incorporating additional biomarkers and imaging techniques could provide a more detailed and nuanced understanding of how EV therapy influences periodontal regeneration and inflammation resolution. In this study, we primarily focused on clinical parameters such as plaque index, bleeding on probing, probing depth, and attachment level to assess the efficacy of EV therapy in the treatment of periodontitis. While these measures are valuable for evaluating the clinical state of periodontal health, they do not provide a complete picture of the morphological changes within the tissue. We recognize this as a limitation of our study, as comprehensive morphological evaluations, such as histological analysis or advanced imaging techniques, such as cone-beam computed tomography [56], could offer deeper insights into the structural restoration of periodontal tissues. These additional methodologies could help in further substantiating the regenerative capabilities of EV therapy, which were inferred from improvements in clinical metrics and reductions in cytokine levels.

To further substantiate the findings and advance our understanding of EV therapy in periodontitis, future research should focus on elucidating the specific mechanisms by which EVs modulate the immune response and promote tissue regeneration. This may involve investigating the molecular cargo carried by exosomes and other vesicles, such as proteins, lipids, and miRNAs, their interactions with target cells, and the consequent alterations in signaling pathways. Understanding these mechanisms in detail will validate the therapeutic potential of EVs and enable the development of more targeted and effective EV-based therapies for periodontitis and other inflammatory diseases. Conducting larger clinical trials with extended follow-up durations and well-defined demographic and comorbidities patient stratification will be crucial to assessing the therapy’s long-term safety and effectiveness. Addressing these limitations in future research is essential for advancing our comprehension of EV therapy’s potential and establishing it as a validated treatment option for periodontitis. The successful confirmation of the biological effect of the therapy through the clinical results obtained, despite the limitations, underscores the value of this proof-of-concept study, and paves the way for further, more detailed investigations.

#### Unseen Risks: The Shadow Side of EV Therapy

EV therapy is a promising treatment approach in regenerative medicine, with many studies reporting minimal or no side effects, such as transient redness at the application site. However, as with any emerging medical therapy, it is crucial to understand and mitigate potential risks to ensure safe and effective application [57].

EVs are generally biocompatible and display lower immunogenicity compared to whole cells. However, EVs can provoke immune responses, especially when derived from allogeneic donors. This categorizes immune reactions as a notable concern.

EVs play a crucial role in cell communication. They can carry growth factors that could inadvertently promote tumor growth or cancer progression, particularly if the EVs originate from malignant cells or contain oncogenic factors. One therapeutic aim of EV therapy is to modulate inflammation, but if not adequately controlled or targeted, EVs might exacerbate inflammation instead of reducing it, influenced by their source and the molecules they transport.

Determining the optimal dosage and most effective administration route for EV therapy can be complex. Inaccurate dosing or delivery methods could reduce therapeutic efficacy or lead to unintended side effects. Manufacturing clinical-grade EVs consistently, safely, and on a scalable level presents significant challenges. Variability in EV preparation can influence the reproducibility of therapeutic outcomes and complicate the regulatory approvals.

EVs naturally carry diverse molecules, including proteins, lipids, and nucleic acids, posing a risk of transferring undesirable molecules alongside therapeutic agents, potentially leading to off-target effects [58]. Inadequately screened and processed source materials can be a source of pathogens transmitted via EVs.

The development, production, and administration costs associated with EV therapy can be substantial, potentially limiting its accessibility to a broader patient population. As EV therapy is relatively novel, the long-term effects and potential risks are not fully understood, emphasizing the need for continuous research and thorough clinical monitoring. Ongoing investigation will help uncover more about EV therapy’s long-term safety and effectiveness, guiding future applications and improvements.

## 4. Materials and Methods

### 4.1. Patient Selection and Evaluation

The study included 14 subjects, comprising eight males and six females, half healthy subjects and half with diagnosed periodontitis at stages I–III. Prior to participation, each individual provided informed consent. A certified periodontologist conducted the initial clinical evaluations. Participants were selected based on their medical history and a clinical examination during their initial visit. The diagnosis of generalized periodontitis at stages I–III was determined according to established criteria: the percentage of teeth affected by the disease, the level of epithelial attachment loss, and the presence of bleeding upon provocation [59].

Seven healthy participants, four males and three females, with no sign of any oral disease, including periodontitis, served as a comparison group to evaluate the efficacy of EV therapy in patients with periodontitis. These healthy individuals served as a baseline for normal cytokine levels, against which the post-treatment levels of patients with periodontitis were compared.

#### 4.1.1. Inclusion Criteria

Patients: Participants eligible for inclusion in the study met the following criteria: diagnosis of generalized periodontitis at stages I–III, aged 18 years or older, systemically healthy, non-smokers, possessing at least 20 natural teeth, and having not undergone any periodontal treatments within the past 6 months. Additionally, they had not used antibiotics, anti-inflammatory drugs, vitamin supplements, or undergone immunosuppressive therapy within the preceding three months.

Healthy participants: Participants eligible for inclusion in the study met the following criteria: no oral pathology, over 18 years old, systemically healthy, non-smokers, possessing at least 20 natural teeth, and having not undergone any periodontal treatments within the past 6 months. Additionally, they had not used antibiotics, anti-inflammatory drugs, vitamin supplements, or undergone immunosuppressive therapy within the preceding three months.

#### 4.1.2. Exclusion Criteria for the Study

Patients: Potential participants were excluded from the study if they met any of the following criteria: diagnosis of generalized periodontitis other than stages I–III; other oral diseases; less than 18 years old; diagnosed with chronic systemic diseases; had fewer than 20 teeth; were smokers; had undergone a periodontal procedure within the last six months; had received antibiotic therapy within the last six months; had taken anti-inflammatory drugs, vitamin supplements, or followed a special diet within the last 3 months; and pregnant or lactating women.

Healthy participants: Potential participants were excluded from the study if they met any of the following criteria: diagnosis of any oral pathology, under the age of 18 years; diagnosed with chronic systemic diseases; had fewer than 20 teeth; were smokers; had undergone a periodontal procedure within the last six months; had received antibiotic therapy within the last six months; had taken anti-inflammatory drugs, vitamin supplements, or followed a special diet within the last three months; and pregnant or lactating women.

### 4.2. The Clinical Validation

Various clinical parameters were used to assess oral hygiene and determine the clinical status of the periodontal tissues. These included the plaque index, gingival index as defined by Löe and Silness [60,61], bleeding on provocation, probing depth, level of epithelial attachment, and level of the gingival margin.

All measurements were performed on all existing teeth with a graduated periodontal probe (PCPUNC 15, Hu Friedy, Chicago, IL, USA). The probe was calibrated twice to maintain inter-researcher consistency and ensure reproducible measurements: once at the outset and at the follow-up. The measurement consistency was confirmed by achieving a Kohen Kappa coefficient greater than 0.75 for each measured parameter.

Clinical measurements and the collection of GCF were conducted during the initial examination before EVs application and then repeated six weeks after the EVs application.

#### Sampling of GCF

Sampling sites for GCF were selected based on anamnesis and clinical examination. GCF samples were taken from two representative sites using filter papers PerioPaper^®^ (Pro-Flow Corp., Amityville, NY, USA) 24 h after measuring and recording clinical parameters, to avoid blood contact immediately after probing.

### 4.3. Preparation of EVs Activated Plasma

The preparation of EV-activated plasma involves size-based separation using the ultracentrifugation method, which effectively retains small and large EVs while removing larger particles and cell debris. Initially, blood samples were subjected to differential centrifugation at 1200× *g* for 15 min at room temperature (RT, approximately 25 °C), and then prolonged at 1500× *g* for 15 min at RT to pellet larger debris and reduce platelet contamination, selectively retaining a supernatant rich in miscellaneous extracellular vesicles (EVs), including exosomes. This supernatant was transferred in stages to new tubes for an extended centrifugation at 110,000× *g* for 120 min at 4 °C, aiming to concentrate the EVs into a pellet [62].

The pellet containing EVs was resuspended in 1 mL of sterile physiological saline.

Quality assessment of the EV preparation was conducted using NanoSight Nanoparticle Tracking Analysis (NTA) technology, specifically the NanoSight NS300 system (Malvern Panalytical, Almelo, The Netherlands). This analysis confirmed the presence of 4.0 × 10^9^/mL EVs measuring 191 nm, 1.2 × 10^9^/mL EVs measuring 361 nm, and 0.8 × 10^9^/mL EVs measuring 385 nm. The distribution suggests a 2:1 ratio of small-sized to large-sized EVs.

Given that the EVs clearly originate from diverse cellular sources, we conducted immune-phenotype analysis. We were cognizant that cytometers are unable to detect EVs ranging in size from 150–250 nm. Having identified larger-sized EVs exceeding 350 nm, we focused our analysis on the CD9-positive EV population, detecting the presence of CD62E (endothelial origin), CD235a (erythrocyte origin), and CD45 (leukocyte origin).

The prepared EVs plasma was administered aseptically in a single-dose, with a total volume of 0.8 mL and a total concentration of 6.0 × 10^9^/mL EVs.

### 4.4. Cytokine Selection

We selected cytokines known to influence inflammatory processes and enhance the immunomodulatory influence for oral health; see Table 11 [20,63]. Note that the classification of cytokines as pro-inflammatory or anti-inflammatory is somewhat simplified in this context, and their roles in oral health are subjects of ongoing research. Many cytokines can exhibit dual roles, depending on the environment and the presence of other cytokines and factors. For instance, TGF-β has both anti-inflammatory and regulatory functions that can contribute to tissue repair, but also to fibrosis under certain conditions.

### 4.5. Statistical Analysis

In this study, a variety of statistical tools and tests were employed to analyze the data collected, ensuring a comprehensive understanding of the results. Here is an overview of these tools and their applications:

1. The Shapiro–Wilk Test assesses the normality of the data’s distribution, which is a prerequisite for many other statistical tests. A *p*-value of less than 0.05 typically indicates that the data do not follow a normal distribution;
2. The Mann–Whitney U Test is a non-parametric test that compares differences between two independent groups when the dependent variable is ordinal or continuous but not normally distributed;
3. A paired *t*-test compares the means of two related groups to determine if there is a statistically significant difference between them. It requires the data to be normally distributed, and is often used to compare the outcome measures before and after an intervention in the same subjects;
4. The Kohen Kappa Coefficient measures the agreement between two raters (or instruments) for categorical outcomes. A Kappa value of 1 indicates perfect agreement, whereas 0 indicates no agreement beyond chance. A Kappa value greater than 0.75 is considered an excellent agreement;
5. NanoSight Nanoparticle Tracking Analysis (NTA): While not a statistical test, the NanoSight NTA system is a technological tool used to characterize nanoparticles, such as EVs, in a liquid suspension. NTA provides information on their size distribution and concentration by analyzing their Brownian motion;
6. Statistical software: The data analysis was conducted using the statistical software package G Power 3.0.10, Statistics Kingdom [79], and Excel tables. These tools offer comprehensive functions for performing statistical tests and analyses, and visualizing the data through graphs and charts for easier interpretation.

## 5. Conclusions

### 5.1. The Significance

This study represents a landmark achievement in the field of periodontal therapy, marking the first published result of a clinical pilot investigating the application of EV therapy for the treatment of periodontitis. Prior to this pioneering work, the potential of EVs in addressing periodontal disease was primarily explored through animal models and reviews of potential clinical impact based on results from other disciplines and periodontal animal models. These preliminary investigations laid the groundwork for understanding the therapy’s mechanisms of action and potential benefits in regenerative medicine. However, the translation of these findings from animal models to human clinical settings remained uncharted territory, providing only review-based points until now. Our study bridges this gap and sets a precedent for future research in this area. By demonstrating significant clinical improvements and offering insights into the immunomodulatory effects of EV therapy in human participants, this research opens new avenues for the development of advanced therapeutic strategies against periodontitis. As a proof-of-concept study, it is a crucial step forward from the theoretical and experimental stages towards practical clinical applications, providing crucial biological evidence of the EV treatment strategy for periodontitis patients, a foundation upon which future studies can build to further refine and enhance the efficacy of the therapy for periodontal and other oral diseases.

### 5.2. Future Research Directions

While EV therapy is still in the exploratory stages for the treatment of periodontitis, the rationale for its use is compelling and supported by the unique properties and potential benefits the therapy. The study provides promising evidence for the therapy’s potential anti-inflammatory and regenerative effects in treating periodontitis. The observed reductions in pro-inflammatory cytokine levels suggest a modulation of the immune response, which may contribute to the attenuation of tissue damage and the promotion of regenerative processes. However, further research is needed to fully understand the underlying mechanisms and to establish EV therapy as a viable therapeutic option for periodontitis and other inflammatory conditions; see Table 12. Suggested future research directions may include:

- Long-term effects of EV therapy: Future studies should prioritize longitudinal designs to assess the durability of the therapy’s benefits over time. This includes monitoring the stability of clinical improvements, such as reductions in probing depth and attachment level gains and the persistence of reduced cytokine levels. Long-term follow-up will help determine if repeated administrations are necessary and evaluate the potential for recurrence of periodontal disease after treatment cessation;
- Mechanisms of action: While the current study highlights the potential of the therapy in modulating immune responses and promoting tissue regeneration, the exact mechanisms remain to be fully elucidated. Future research should leverage advanced molecular and cellular biology techniques, such as transcriptomic and proteomic analyses, to unravel the specific pathways influenced by EV therapy. Understanding how EVs interact with periodontal cells and tissues at the molecular level will enable the refinement of therapy to enhance its efficacy and specificity;
- Larger-scale clinical trials: Future research should include multi-center, randomized controlled trials with larger participant cohorts to overcome the limitations posed by small sample sizes and single-center studies. Such studies would provide more robust evidence of the therapy’s effectiveness and safety, facilitating the generalization of the findings to a broader population. Moreover, stratifying participants based on periodontitis severity, genetic predispositions, and other relevant factors could uncover differential responses to treatment, guiding personalized therapeutic approaches;
- Standardization of EV preparations: Given the potential variability in EVs characteristics based on their source and the extraction method, establishing standardized protocols for their preparation is critical. Future research should aim to characterize and standardize the dose, purity, and composition of EVs preparations to ensure consistent therapeutic outcomes. Collaborative efforts to develop and validate standardized EV products could accelerate their clinical application;
- Comparative studies: Investigating how EV therapy compares to or synergizes with existing periodontal treatments could provide valuable insights into its relative effectiveness and potential role within the current treatment paradigm. Studies comparing EV therapy alone, in combination with traditional treatments, such as scaling and root planing, and in comparison to other regenerative approaches, like growth factor applications and tissue engineering strategies, would be informative;
- Exploration of biomarkers for treatment monitoring: Identifying specific biomarkers that can predict response to EV therapy or monitor its progression could enhance the precision of treatment. Research into the correlation between specific cytokines, genetic markers, or other molecular indicators with treatment outcomes could facilitate the development of personalized treatment plans and real-time therapeutic efficacy monitoring;
- Detailed morphological assessments: Histological examinations would provide cellular-level insights into the regeneration of periodontal tissues, potentially correlating the observed clinical improvements with histopathological changes. Additionally, advanced imaging techniques such as micro-CT or MRI could be employed to visualize and quantify the structural changes in the alveolar bone and surrounding periodontal ligament. These methods would enhance our understanding of the mechanisms behind the observed clinical benefits, and provide comprehensive validation of the therapy’s regenerative impact.

By addressing these specific areas, future research can build upon the foundational knowledge established by initial studies, driving forward the development of EV therapy as a sophisticated, targeted treatment strategy for periodontitis and potentially other inflammatory and regenerative conditions. The significant differences observed before treatment underscore the importance of targeted therapy that can specifically address these altered cytokine levels. EV therapy is a promising approach in this direction, as indicated by the normalization of some cytokine levels post-treatment. Understanding the cytokine profile of healthy individuals versus those with periodontitis, both pre- and post-EV therapy, can enhance our understanding of the disease mechanism. This knowledge can drive the development of targeted therapies and personalized medicine approaches. Further research with larger sample sizes and additional cytokines might provide more insights into the specific effects and mechanisms of action of EV therapy in periodontitis.

#### 5.2.1. Systemic Implications

The potential of EV therapy to modulate cytokine levels in periodontitis has implications that extend beyond just oral health. Periodontitis is increasingly recognized as a multidimensional inflammatory condition with systemic links to several chronic diseases. Many of the cytokines dysregulated in periodontitis, such as IL-6, TNF-α, and IL-17A, play pathogenic roles in conditions like cardiovascular disease, rheumatoid arthritis, diabetes, stress, and certain cancers. Reducing the levels of these inflammatory mediators in the periodontal environment through EV therapy may mitigate their systemic spillover effects [80,81,82,83]. This could help disrupt the pathogenic crosstalk between oral and systemic inflammation that contributes to the initiation and progression of associated comorbidities. Additionally, exosomes can also carry and deliver immunomodulatory cargo that could influence systemic inflammatory processes [84,85]. As such, applying EV therapy for periodontitis may have far-reaching impacts beyond just oral health by targeting shared inflammatory mechanisms underlying various systemic diseases. This underscores the potential of EV-based approaches to bridge the gap between periodontal medicine and overall systemic health and well-being.

#### 5.2.2. Comparative Efficacy and Integration of EV therapy in Periodontal Treatment

EV therapy shows promising results in the management of periodontitis, but its efficacy and practicality should be evaluated against existing therapies, such as scaling and root planing, and advanced surgical interventions. While various regenerative medicine approaches offer new hope for patients with advanced periodontitis, the comprehensive benefits of EV therapy—ranging from biocompatibility and safety to its less invasive nature and potential for customization—underscore its role as a leading treatment option in this challenging field. Future research should include comparative studies that benchmark the outcomes of EV therapy against these traditional and novel regenerative treatments and assess the cost-effectiveness, patient compliance, and long-term benefits.

However, understanding how EV therapy can be integrated into existing treatment protocols could significantly enhance periodontal disease management, potentially shifting the current therapeutic paradigm towards minimally invasive options. The broader implications of this therapy extend beyond dental health, as reducing systemic inflammation associated with periodontitis could also mitigate its links to cardiovascular diseases, diabetes, and other systemic conditions. Despite these considerations, the findings of this study represent a significant step towards developing innovative and potentially transformative therapeutic approaches for periodontitis, with EV therapy emerging as a promising candidate for addressing the limitations of conventional treatment modalities.

## Figures and Tables

**Table 1:** Periodontitis stages. This classification is based on the 2017 World Workshop on the Classification of Periodontal and Peri-Implant Diseases and Conditions [6]. The classification system outlines the stages of periodontitis based on the severity, complexity, and extent of the disease, providing a framework for diagnosis and treatment planning.

Stage	Description	Severity	Complexity
Stage I	Initial periodontitis	Mild	Least complex; primarily interdental CAL of 1–2 mm, no tooth loss, mostly horizontal bone loss.
Stage II	Moderate periodontitis	Moderate	Increased CAL of 3–4 mm, no tooth loss, mostly horizontal bone loss.
Stage III	Severe periodontitis with potential for additional tooth loss	Severe	CAL ≥ 5 mm, tooth loss due to periodontitis (≤4 teeth), vertical bone loss ≥ 3 mm or furcation involvement Class II/III, moderate ridge defect.
Stage IV	Severe periodontitis with extensive tooth loss and potential for loss of dentition	Very severe	CAL ≥ 5 mm, tooth loss due to periodontitis (>4 teeth), need for complex rehabilitation, masticatory dysfunction, bite collapse, drifting, flaring.

CAL: Clinical Attachment Loss; Interdental CAL: Clinical Attachment Loss between teeth; Horizontal bone loss: loss of bone parallel to the cementoenamel junction; Vertical bone loss: loss of bone in a vertical direction leading to deep pockets.

**Table 2:** Demographics and clinical characteristics (mean ± standard deviation (SD)) from baseline measurements of active (before the treatment) and inactive sites (6 weeks after the therapy).

Clinical Parameters	Active Sites (*n* = 14) (Before Treatment)	Inactive Sites (*n* = 14) (After Treatment)
Mean probing depth (mm) ± SD	5.42 ± 0.46	4.14 ± 0.62
Mean attachment level (mm) ± SD	6.20 ± 0.76	5.42 ± 0.68
% sites with plaque	100	10
% sites with bleeding on probing	100	10
Age (years)	47.85 ± 6.32 (34–65)	
Males (%)	57.14 (4/7)	

**Table 3:** Descriptive statistics of cytokine levels in GCF in active sites (before treatment).

Cytokine	Mean	Std Dev	Min	Max	Skewness	Kurtosis	Normal Dist
IL5	9.58	0.83	8.83	11.16	1.38	1.52	True
IL6	2.30	0.60	1.66	3.13	0.75	−1.20	True
IL17A	42.42	2.74	39.63	47.02	0.85	−0.33	True
IL22	89.30	28.09	52.25	120.50	−0.10	−2.11	True
IL21	8.81	1.69	7.40	11.60	0.78	−1.06	True
IL10	27.70	12.91	15.50	42.00	0.33	−2.73	False
IL1β	26.65	12.85	17.86	47.21	1.25	−0.67	False
IL13	12.65	4.18	7.66	21.15	1.52	3.61	True
TNFα	9.01	5.06	3.51	15.69	0.44	−1.37	True
IL27	3.87	0.77	2.67	4.68	−0.97	−0.80	True
IL2	6.66	1.80	5.15	10.00	1.36	0.87	True
IFNγ	12.17	4.00	6.64	16.29	−0.65	−1.53	True
IL9	9.30	1.93	7.45	12.08	0.51	−2.05	True
IL4	8.48	3.40	5.59	15.96	2.32	5.88	False
IL12	7.20	1.08	6.12	8.88	0.48	−1.19	True
GMCSF	4.98	0.73	3.97	5.98	−0.07	−1.48	True

**Table 4:** Descriptive statistics of cytokine levels in GCF in inactive sites (6 weeks after treatment).

Cytokine	Mean	Std Dev	Min	Max	Skewness	Kurtosis	Normal Dist
IL5	4.13	1.92	3.16	8.45	2.54	6.57	False
IL6	0.84	0.59	0.38	2.01	1.60	2.15	False
IL17A	34.13	13.79	3.51	44.33	−2.42	6.13	False
IL22	70.50	28.63	50.64	116.48	1.23	−0.66	False
IL21	5.45	1.49	4.02	7.80	1.09	−0.72	False
IL10	11.24	5.97	4.86	18.42	−0.08	−2.43	True
IL1β	18.74	11.09	12.14	43.69	2.56	6.66	False
IL13	9.30	2.11	7.04	11.28	−0.26	−2.68	False
TNFα	2.98	0.42	2.16	3.47	−1.35	2.61	True
IL27	3.86	0.97	2.29	4.92	−0.92	−0.61	True
IL2	6.66	1.49	4.93	8.62	0.50	−1.70	True
IFNγ	6.36	0.75	5.32	7.46	−0.06	−0.70	True
IL9	9.31	2.49	6.46	13.03	0.54	−1.21	True
IL4	6.86	2.88	3.73	12.37	1.06	2.12	True
IL12	4.62	1.62	2.17	6.94	−0.02	−0.64	True
GMCSF	4.25	0.91	3.41	6.10	1.61	3.19	True

**Table 5:** Paired statistical test results for cytokine levels (before vs. after treatment). A *p*-value of less than 0.05 indicates the statistical significance. See text for details. The choice of statistical tests was based on the normality of the distributions, ensuring appropriate analysis for each cytokine.

Cytokine	*p*-Value	Test Type
IL5	▼ 0.0156	Mann–Whitney U
IL6	▼ 0.0313	Mann–Whitney U
IL17A	▼ 0.0469	Mann–Whitney U
IL22	0.0781	Mann–Whitney U
IL21	▼ 0.0469	Mann–Whitney U
IL10	▼ 0.0156	Mann–Whitney U
IL1β	0.1563	Mann–Whitney U
IL13	0.2188	Mann–Whitney U
TNFα	▼ 0.0235	*t*-test
IL27	0.9785	*t*-test
IL2	0.9961	*t*-test
IFNγ	▼ 0.0121	*t*-test
IL9	0.9921	*t*-test
IL4	0.2188	Mann–Whitney U
IL12	▼ 0.0305	*t*-test
GMCSF	0.1328	*t*-test

▼ Denotes a significant decrease in the cytokine level.

**Table 6:** Levels of selected cytokines before and after the EV treatment per each patient (total amount pg).

Cytokine	Condition	Patient 1	Patient 2	Patient 3	Patient 4	Patient 5	Patient 6	Patient 7
IL5	Before	8.83	8.92	9.32	11.16	9.54	9.11	10.15
	After	3.22	3.51	3.92	3.48	8.45	3.16	3.2
IL6	Before	2.23	1.86	1.66	3.13	2.23	1.87	3.11
	After	0.38	0.41	2.01	0.56	1.22	0.48	0.79
IL17A	Before	39.63	47.02	41.91	42.31	45.13	41.16	39.79
	After	3.51	39.22	44.33	37.61	40.3	38.78	35.16
IL21	Before	7.74	7.4	7.6	11.6	7.4	9.9	10.05
	After	4.71	7.36	7.8	4.52	4.93	4.78	4.02
IL10	Before	20.24	41.04	42	41.2	17.52	16.4	15.5
	After	4.86	5.26	18.42	5.25	16.67	15.5	12.7
TNFα	Before	15.56	7.7	3.53	15.69	3.51	7.15	9.9
	After	2.83	3.05	3.21	2.95	3.47	3.2	2.16
IFNγ	Before	7	13.87	6.64	14.63	16.29	15.57	11.16
	After	6.6	6.22	7.46	6.42	6.94	5.54	5.32
IL12	Before	7.55	7.21	6.12	6.34	6.14	8.18	8.88
	After	3.87	3.95	6.94	3.87	5.77	5.79	2.17

**Table 7:** Descriptive statistics of cytokine levels in GCF of healthy subjects.

Cytokine	Mean	Std Dev	Min	Max	Skewness	Kurtosis	Normal Dist
IL5	3.84	1.95	2.70	8.23	1.98	2.04	False
IL6	0.71	0.58	0.25	1.87	1.29	0.32	False
IL17A	32.54	13.41	2.72	42.25	−1.87	1.82	False
IL22	66.90	29.64	45.24	114.25	0.93	−1.04	False
IL21	4.99	1.58	3.52	7.77	0.97	−0.69	False
IL10	9.87	5.08	4.11	16.44	0.03	−1.67	True
IL1b	17.61	11.12	12.02	42.78	2.02	2.13	False
IL13	8.49	2.31	5.02	10.54	−0.45	−1.55	False
TNFα	2.78	0.51	2.03	3.36	−0.44	−1.27	True
IL27	3.36	1.15	1.46	4.64	−0.67	−0.94	True
IL2	5.98	1.61	4.36	8.24	0.33	−1.62	True
IFNγ	5.87	0.68	5.12	7.07	0.51	−0.57	True
IL9	8.50	2.17	6.14	11.98	0.49	−1.09	True
IL4	6.26	2.71	3.02	11.54	0.97	0.27	True
IL12	4.06	1.38	2.09	6.14	0.11	−1.06	True
GMCSF	3.57	1.22	2.21	5.74	0.56	−0.49	True

**Table 8:** Cytokine comparison between the healthy subjects and subjects with periodontitis stages I–III before the treatment. The blue triangle denotes a significant decrease in the cytokine level.

Cytokine	*p*-Value	Test Type
IL5	▼ 0.0006	Mann–Whitney U
IL6	▼ 0.0059	Mann–Whitney U
IL17A	▼ 0.0070	Mann–Whitney U
IL22	0.0728	Mann–Whitney U
IL21	▼ 0.0105	Mann–Whitney U
IL10	▼ 0.0023	Mann–Whitney U
IL1β	▼ 0.0111	Mann–Whitney U
IL13	▼ 0.0297	Mann–Whitney U
TNFα	▼ 0.0071	*t*-test
IL27	0.3458	*t*-test
IL2	0.4694	*t*-test
IFNγ	▼ 0.0015	*t*-test
IL9	0.4833	*t*-test
IL4	0.0550	Mann–Whitney U
IL12	▼ 0.0005	*t*-test
GMCSF	▼ 0.0226	*t*-test

▼ denotes a significant decrease in the cytokine level.

**Table 9:** Cytokine comparison between the healthy subjects and subjects with periodontitis stages I–III six weeks post-treatment.

Cytokine	*p*-Value	Test Type
IL5	0.124	Mann–Whitney U
IL6	0.406	Mann–Whitney U
IL17A	0.456	Mann–Whitney U
IL22	0.259	Mann–Whitney U
IL21	0.259	Mann–Whitney U
IL10	0.653	*t*-test
IL1b	0.097	Mann–Whitney U
IL13	0.250	Mann–Whitney U
TNFα	0.430	*t*-test
IL27	0.395	*t*-test
IL2	0.430	*t*-test
IFNγ	0.228	*t*-test
IL9	0.529	*t*-test
IL4	0.695	*t*-test
IL12	0.500	*t*-test
GMCSF	0.259	*t*-test

**Table 10:** Summary of key findings.

Key Finding	Significance
Significant reduction in pro-inflammatory cytokines	Indicates a substantial anti-inflammatory impact of the therapy, implying its effectiveness in interrupting the pathological inflammatory responses associated with periodontitis.
Improvement in clinical parameters (e.g., probing depth, attachment level)	Demonstrates the therapy’s ability not only to arrest the progression of the disease, but also to facilitate the regeneration of periodontal tissues, thereby improving overall gum health.
No adverse effects reported	The lack of reported negative effects underscores the therapy’s favorable safety profile, making it a suitable alternative for patients desiring minimal-risk treatments.
Convergence of cytokine levels towards healthy baselines post-treatment	The observed alignment of cytokine levels after treatment with those of healthy individuals underscores a noteworthy shift toward a normal immune response, demonstrating the therapy’s role in alleviating periodontal inflammation and promoting immune health. This dual action of EV therapy provides a compelling approach to comprehensive periodontal disease management and recovery.

**Table 11:** Cytokines: inflammatory classification and roles in oral health. A snapshot of various cytokines’ complex interactions and roles in oral health, particularly in periodontitis. Note that the inflammatory classification of some cytokines can vary depending on the context, and their roles in oral health are subjects of ongoing research.

Cytokine	Inflammatory Classification	Roles in Oral Health
IL-5	Pro-inflammatory	Involved in eosinophil activation and has been associated with allergic responses [25].
IL-6	Pro-inflammatory	Facilitates the transition from acute to chronic inflammation, promoting periodontitis progression. It also influences bone metabolism, and can induce osteoclast formation [26,27].
IL-17A	Pro-inflammatory	Promotes inflammation and bone resorption in periodontitis by stimulating the production of pro-inflammatory cytokines and matrix metalloproteinases [29,30].
IL-22	Regulatory	Plays a role in mucosal defense mechanisms and healing processes. Its exact role in oral health is under investigation [64,65].
IL-21	Pro-inflammatory	Supports the inflammatory response, and has been implicated in autoimmune diseases, suggesting a potential role in inflammatory oral conditions [66,67].
IL-10	Anti-inflammatory	Limits immune responses and inflammation, protecting against excessive tissue damage in periodontal disease [31,32].
IL-1β	Pro-inflammatory	Promotes bone resorption, and is a key player in the inflammatory response to periodontal pathogens, stimulates osteoclastogenesis, and contributes to tissue destruction in periodontitis [33,68].
IL-13	Anti-inflammatory	Inhibits inflammatory cytokine production, potentially playing a protective role against periodontal disease [69].
TNF-α	Pro-inflammatory	Significant in the destruction of periodontal tissues, stimulates other pro-inflammatory cytokines’ production, and contributes to bone resorption [33,34].
IL-27	Regulatory/Anti-inflammatory	Involved in regulating immune responses, and has been shown to have anti-inflammatory effects, potentially protective in periodontitis [70,71].
IL-2	Pro-inflammatory	Promotes T-cell growth and differentiation; its role in oral health might relate to its effects on cellular immunity within the periodontal environment [72].
IFN-γ	Pro-inflammatory	Has antimicrobial effects, and modulates the immune response by activating macrophages and promoting Th1 immune responses, potentially exacerbating chronic periodontal inflammation [73].
IL-9	Pro-inflammatory	Involved in mast cell activation, and may contribute to inflammatory responses, though its specific role in oral health requires further study [74].
IL-4	Anti-inflammatory	Modulates inflammatory responses, promoting antibody responses to suppress pro-inflammatory cytokines’ production. This might play a protective role in periodontal health and inhibit bone resorption [12,75].
IL-12	Pro-inflammatory	Induces the production of IFN-γ, promoting Th1 responses. Its role in oral health might involve modulating immune responses to periodontal pathogens [76,77].
GM-CSF	Pro-inflammatory	Enhances neutrophil function, and is involved in the defense against pathogens, potentially playing a role in the protection and exacerbation of periodontal disease [78].

**Table 12:** Summary of future research directions.

Future Research Direction	Objective/Purpose/Goal	Rationale
Longitudinal clinical trials	To evaluate the long-term efficacy and safety of EV therapy in periodontitis.	To provide insights into the durability of treatment effects, potential for recurrence, and long-term safety profile, which are essential for translating EV therapy into routine clinical practice.
Mechanistic studies	To explore the detailed molecular mechanisms of action of EV therapy.	Understanding the precise biological pathways and molecular interactions mediated by EVs will facilitate the optimization of therapy and identification of the biomarkers for treatment monitoring and outcome prediction.
Larger-scale, multicenter trials	To assess the effectiveness of EV therapy across diverse populations and settings.	Conducting studies with larger participant cohorts and across multiple centers will enhance the generalizability of findings and validate the therapy’s effectiveness and safety on a broader scale.
Standardization and optimization of EV preparations	To develop standardized protocols for preparing, characterizing, and dosing EV therapies.	Standardizing EVs production will ensure therapeutic outcomes’ consistency, quality, and reproducibility. It is crucial for regulatory approval and clinical adoption of EVs-based treatments.
Comparative efficacy studies	To compare EV therapy with existing periodontal treatments and other regenerative therapies.	Comparative studies will elucidate the relative effectiveness and potential synergies between EV therapy and current treatment modalities, informing clinical decision-making and treatment optimization.
Personalized therapy approaches	To investigate the potential for personalized EV therapies tailored to individual patient needs and disease profiles.	Personalized medicine approaches, leveraging patient-specific characteristics and biomarkers, could optimize treatment efficacy and minimize side effects, paving the way for targeted and individualized therapeutic strategies.
Exploration of periodontitis EV therapy in other diseases	To investigate the applicability of EV therapy in treating other inflammatory and regenerative conditions beyond periodontitis.	EVs’ anti-inflammatory and regenerative properties hold promise for a wide range of conditions. Expanding research to comorbidities could uncover new therapeutic applications and benefits of the therapy.

## Data Availability

The datasets collected during or analyzed during the current study are available from the corresponding author upon reasonable request.
